# Development and Validation of Esophageal Squamous Cell Carcinoma Risk Prediction Models Based on an Endoscopic Screening Program

**DOI:** 10.1001/jamanetworkopen.2022.53148

**Published:** 2023-01-26

**Authors:** Junming Han, Xiaolei Guo, Li Zhao, Huan Zhang, Siqi Ma, Yan Li, Deli Zhao, Jialin Wang, Fuzhong Xue

**Affiliations:** 1 Department of Biostatistics, School of Public Health, Cheeloo College of Medicine, Shandong University, Jinan, China; 2 Healthcare Big Data Research Institute, School of Public Health, Cheeloo College of Medicine, Shandong University, Jinan, China; 3 The Department for Chronic and Noncommunicable Disease Control and Prevention, Shandong Center for Disease Control and Prevention and Academy of Preventive Medicine, Shandong University, Jinan, China; 4 Department of Scientific Research and Teaching, Feicheng Hospital Affiliated to Shandong First Medical University, Feicheng, China; 5 School of Public Health, Shandong First Medical University and Shandong Academy of Medical Sciences, Jinan, China; 6 Cancer Prevention and Treatment Center, Feicheng People’s Hospital, Feicheng, China; 7 Department of Human Resource, Shandong Cancer Hospital and Institute, Shandong First Medical University and Shandong Academy of Medical Sciences, Jinan, China; 8 Qilu Hospital, Cheeloo College of Medicine, Shandong University, Jinan, China

## Abstract

**Importance:**

Assessment tools are lacking for screening of esophageal squamous cell cancer (ESCC) in China, especially for the follow-up stage. Risk prediction to optimize the screening procedure is urgently needed.

**Objective:**

To develop and validate ESCC prediction models for identifying people at high risk for follow-up decision-making.

**Design, Setting, and Participants:**

This open, prospective multicenter diagnostic study has been performed since September 1, 2006, in Shandong Province, China. This study used baseline and follow-up data until December 31, 2021. The data were analyzed between April 6 and May 31, 2022. Eligibility criteria consisted of rural residents aged 40 to 69 years who had no contraindications for endoscopy. Among 161 212 eligible participants, those diagnosed with cancer or who had cancer at baseline, did not complete the questionnaire, were younger than 40 years or older than 69 years, or were detected with severe dysplasia or worse lesions were eliminated from the analysis.

**Exposures:**

Risk factors obtained by questionnaire and endoscopy.

**Main Outcomes and Measures:**

Pathological diagnosis of ESCC and confirmation by cancer registry data.

**Results:**

In this diagnostic study of 104 129 participants (56.39% women; mean [SD] age, 54.31 [7.64] years), 59 481 (mean [SD] age, 53.83 [7.64] years; 58.55% women) formed the derivation set while 44 648 (mean [SD] age, 54.95 [7.60] years; 53.51% women) formed the validation set. A total of 252 new cases of ESCC were diagnosed during 424 903.50 person-years of follow-up in the derivation cohort and 61 new cases from 177 094.10 person-years follow-up in the validation cohort. Model A included the covariates age, sex, and number of lesions; model B included age, sex, smoking status, alcohol use status, body mass index, annual household income, history of gastrointestinal tract diseases, consumption of pickled food, number of lesions, distinct lesions, and mild or moderate dysplasia. The Harrell C statistic of model A was 0.80 (95% CI, 0.77-0.83) in the derivation set and 0.90 (95% CI, 0.87-0.93) in the validation set; the Harrell C statistic of model B was 0.83 (95% CI, 0.81-0.86) and 0.91 (95% CI, 0.88-0.95), respectively. The models also had good calibration performance and clinical usefulness.

**Conclusions and Relevance:**

The findings of this diagnostic study suggest that the models developed are suitable for selecting high-risk populations for follow-up decision-making and optimizing the cancer screening process.

## Key Points

**Question:**

How can patients at high risk of developing esophageal squamous cell carcinoma be identified for follow-up after endoscopic screening?

**Findings:**

The risk prediction model developed in this diagnostic study of 104 129 participants had good performance for identifying a high-risk population for follow-up in cancer screening.

**Meaning:**

These findings suggest that for esophageal cancer screening, this risk prediction model could provide useful guidance in follow-up decision-making.

## Teaser

This diagnostic study establishes and validates esophageal squamous cell cancer prediction models for the follow-up stage of esophageal cancer screening and optimization of the esophageal cancer screening program.

## Introduction

Globally, esophageal cancer ranks eighth among common malignant tumors and sixth among cancer-related causes of death.^1,2,3^ In China, the number of new cases of esophageal cancer in 2020 was 324 422 (95% uncertainty interval, 319 432-329 490), while the age-standardized rate was 13.8 per 100 000 population, ranking sixth among common malignant tumors.^4,5,6^ Esophageal adenocarcinoma (EAC) and esophageal squamous cell carcinoma (ESCC) are 2 main histological subtypes of esophageal cancer. Esophageal adenocarcinoma is more common in Western countries with a rising incidence,^7^ while ESCC accounts for over 90% of esophageal cancer cases and is more common in East Africa, Central Asia, and China. Although the incidence of ESCC declined slightly, it is still high.^5,8,9^

Esophageal cancer is an early asymptomatic and highly fatal disease, and most patients are already at an advanced stage when diagnosed. Despite great progress in the relevant treatment therapy, the 5-year survival rate of esophageal cancer is still poor, even in high-income countries (eg, in the United Kingdom, the 5-year survival rate is lower than 20%).^10,11,12,13^ However, multiple studies have shown that patients with early esophageal cancer who receive endoscopic or surgical treatment have a higher 5-year survival rate.^14,15^ Therefore, early diagnosis and treatment are necessary to improve the prognosis of patients with esophageal cancer. Currently, endoscopy is the main modality used for esophageal cancer screening.^10,11^ Because of the geographic distribution of esophageal cancer, the gastroenterology societies of the United Kingdom and the US focus more on Barrett esophagus and EAC screening, which is not cost efficient in China.^16^ In 2005, the Chinese government issued guidelines for the early diagnosis and treatment of cancer.^12,17^ These guidelines state that esophageal cancer screening is suitable in rural and urban high-risk areas.

According to the screening program (eFigure 1 in Supplement 1), the treatment or follow-up suggestions are mainly for individuals diagnosed with dysplasia or worse lesions, while other groups have no corresponding follow-up or health guidance.^8^ The development direction of esophageal cancer screening is precision, individualization, and risk prediction based; therefore, the prediction model plays an important role.^11^ Existing prediction models (eTable 1 in Supplement 1) were limited and have many defects.^16,18,19^ Prediction models based on case-control studies have low evidence quality,^20,21,22,23^ and cohort-based models rarely perform external validation, tend to be based on questionnaire data, and do not consider endoscopic-related risk factors.^24,25^ Therefore, this report aims to establish and validate ESCC prediction models for the follow-up stage of esophageal cancer screening and to optimize the esophageal cancer screening program.

## Methods

### Study Population

This diagnostic study was based on esophageal cancer screening data in Shandong Province, China. The screening procedure is shown in eFigure 1 in Supplement 1 and has been described in detail previously.^10^ In brief, upper gastrointestinal tract endoscopy and histopathological examination were conducted to detect suspicious lesions in a high-risk population (aged 40-69 years with no contraindications related to endoscopy). During the examination, iodine staining and indicative biopsy were performed. A questionnaire was used to collect information on the esophageal cancer risk factors of participants. All participants signed an informed consent form before endoscopy. All procedures that involved human participants were approved by the Ethics Committee of the Shandong Cancer Hospital and Institute and complied with the ethical standards laid down in the 1964 Declaration of Helsinki as well as its later amendments and comparable ethical standards. This study followed the Transparent Reporting of a Multivariable Prediction Model for Individual Prognosis or Diagnosis (TRIPOD) reporting guideline for diagnostic studies.

The screening data generated by the Feicheng city screening center (September 1, 2006, to February 28, 2019) were used to train our models. The data collected by 15 other centers (January 1, 2017, to August 31, 2020) were used for validation purposes.

A total of 161 212 individuals were assessed for study eligibility. The inclusion criteria used in this study were (1) participation in esophageal cancer screening in Shandong Province since 2006; (2) aged 40 to 69 years; (3) without any cancer, including records by cancer registry or diagnosis at baseline; (4) signed informed consent; (5) completed baseline questionnaire; and (6) no diagnosis with severe dysplasia or worse lesions at baseline. A total of 59 481 individuals formed the derivation cohort after excluding 930 diagnosed with any other cancer at or before baseline, 1084 older than 69 or younger than 40 years, 18 561 with incorrect identification numbers, 1 with incorrect investigation data, 629 diagnosed with severe dysplasia or worse lesions, and 6999 without completed questionnaires. A total of 44 648 individuals formed the validation cohort as shown in eFigure 2 in Supplement 1.

### Candidate Predictors and Data Collection

Multiple types of data (including baseline survey, endoscopy, pathological, cancer registration, and registration of cause of death) were collected. The candidate risk factors for ESCC were selected according to review articles and previous research^3,21,24,25,26,27,28,29,30,31,32^ and were described in detail in eTable 2 in Supplement 1. In addition to the commonly used ESCC risk factors, 3 endoscopy-related factors—number of lesions, distinct lesions, and moderate or mild dysplasia—were also considered in our models. Number of lesions constitutes a categorical factor with 4 levels (0, 1, 2, and ≥3) and is used to measure the number of suspicious lesions or positive findings detected in the esophagus. Distinct lesions constitute a binary factor used to indicate whether there are lesions larger than 1 cm. Moderate or mild dysplasia was derived from the pathological examination results and was divided into 0 (none) and 1 (yes) categories.

### Outcome Definitions

Cases of ESCC were diagnosed according to *International Statistical Classification of Diseases and Related Health Problems, Tenth Revision*, code C15, as well as histology (*International Classification of Diseases for Oncology* morphology codes M8050-M8078 or M8083-M8084) and were confirmed by cancer register record. Pathological diagnosis of biopsy specimens was performed by 2 experienced pathologists blinded to endoscopic findings, and discrepancies in pathological diagnoses were adjudicated by consultation. A pathological quality control expert group was also formed for the esophageal cancer screening. The follow-up of all participants was up to December 31, 2021, for both derivation and validation cohorts.

### Statistical Analysis

The data were analyzed between April 6 and May 31, 2022. Established methods were used to develop and validate ESCC prediction models.^33,34,35^ First, an initial analysis was based on participants with complete data. Then, multiple imputation with chained equations was used to replace the missing values of body mass index (BMI; calculated as weight in kilograms divided by height in meters squared [0.08%]), annual household income (0.02%), source of drinking water (4.42%), smoking status (0.04%), alcohol use status (0.03%), consumption of tea (0.03%), consumption of fresh fruit (1.83%), consumption of pickled food (1.08%), consumption of fried food (4.24%), consumption of hot food (3.78%), history of gastrointestinal tract diseases (0.04%), and family history of any cancer (0.05%). These imputed data sets were used for our main analyses. Five imputed data sets were used, as this approach has a relatively high efficiency. Rubin rules were used to combine the results among the different imputed data sets.^36^

We built 2 models to predict the 3-year ESCC risk. Model A included the 3 variables of age, sex, and number of lesions without selection procedure. The least absolute shrinkage and selection operator (LASSO) method and cross-validation were used to confirm suitable tuning parameters (λ) to determine the predictors in model B.^37^ Interactions between predictors and age were examined in both models, and those that were significant at *P* < .05 were included in the final models. Model B was transformed into a convenient score model.^25,38^ The regression coefficients were used as weights, and the other predictors obtained their corresponding scores by dividing the smallest coefficient and rounding it to an integer. The total score can be calculated according to the prediction factors of each participant, and the risk within 3 years can be calculated.

#### Assessment of Model Performance

We tested model performance by evaluating several indicators. These included *R*^2^ (evaluating the variation the model explained), D statistic (95% CI) (evaluating discriminative ability), Harrell C statistic (95% CI) (evaluating discriminative ability), calibration curve (evaluating the consistence between predicted and observed probability), and decision curve (evaluating clinical usefulness performance).^39,40^

#### External Validation

Multiple imputation was also used to replace missing values in the validation data, and 5 imputed data sets were formed. We tested the performance of the prediction models in validation data. The same evaluation index was used to perform the external validation, and Rubin rules were used to combine the results among the different data sets.

We also performed sensitivity analyses by different groups, age as continuous and categorical variables, BMI in different classification criteria, and deleting participants with dysplasia at baseline. R software, version 4.0.4 (R Project for Statistical Computing) was used for all of the analyses.^41^ The threshold for significance was *P* < .05 in 2-sided tests.

## Results

### Baseline Characteristics

Table 1 shows the characteristics of the 104 129 participants (56.39% women and 43.61% men; mean [SD] age, 54.31 [7.64] years), including 59 481 in the derivation cohort and 44 648 in the validation cohort at baseline. Women accounted for 58.55% and men for 41.45% of participants in derivation data set (mean [SD] age, 53.83 [7.64] years); women accounted for 53.51% and men for 46.49% in the validation data set (mean [SD] age, 54.95 [7.60] years). Participants younger than 50 years accounted for 32.83% in the derivation data set and 27.20% in the validation data set; smokers accounted for 21.28% and 17.16%, respectively; and alcohol users accounted for 22.87% and 17.40%, respectively. Participants found to have lesions accounted for 18.18% in the derivation data set and 16.31% in the validation data set; participants with mild or moderate dysplasia accounted for 5.82% and 2.15%, respectively; and participants with distinct lesions of 1 cm or larger accounted for 4.04% and 4.69%, respectively. Patients with ESCC were more likely to be men and older; to have lower weight, lower fruit intake, and lower annual household income; to be smokers and use alcohol; and to be more likely to have lesions detected by endoscopy at baseline as shown in eTable 3 in Supplement 1. eFigure 3 in Supplement 1 shows the log-rank test of endoscopy-related risk factors. The larger the lesion and the more lesions detected, the greater the cumulative incidence, and we found that number of lesions, size of distinct lesions, and presence of mild or moderate dysplasia were all positively associated with the ESCC risk.

**Table 1.** Baseline Characteristics of Participants at Study Entry in Derivation and Validation Cohorts

Variable	Cohort^a^	
	Derivation (n = 59 481)	Validation (n = 44 648)
Age, mean (SD), y	53.83 (7.64)	54.95 (7.60)
Age, y		
40-44	7757 (13.04)	4093 (9.17)
45-49	11 773 (19.79)	8050 (18.03)
50-54	12 703 (21.36)	10 134 (22.70)
55-59	11 279 (18.96)	7879 (17.65)
60-64	10 069 (16.93)	8518 (19.08)
65-69	5900 (9.92)	5974 (13.38)
Sex		
Female	34 827 (58.55)	23 891 (53.51)
Male	24 654 (41.45)	20 757 (46.49)
Annual household income level		
High	20 339 (34.19)	23 328 (52.25)
Low	39 129 (65.78)	21 320 (47.75)
NA	13 (0.02)	0
Source of drinking water		
Treated	11 792 (19.82)	37 923 (84.94)
Untreated	45 054 (75.75)	4088 (9.16)
NA	2635 (4.43)	2637 (5.91)
Smoking status		
No	46 800 (78.68)	36 986 (82.84)
Yes	12 655 (21.28)	7662 (17.16)
NA	26 (0.04)	0
Alcohol use		
No	45 855 (77.09)	36 879 (82.60)
Yes	13 605 (22.87)	7769 (17.40)
NA	21 (0.04)	0
Consumption of tea		
No	16 433 (27.63)	29 993 (67.18)
Yes	43 027 (72.34)	14 655 (32.82)
NA	21 (0.04)	0
BMI		
≥24	33 713 (56.68)	23 622 (52.91)
<24	25 720 (43.24)	21 026 (47.09)
NA	48 (0.08)	0
Consumption of fresh fruit		
High level	40 676 (68.38)	33 155 (74.26)
Low level	17 714 (29.78)	11 493 (25.74)
NA	1091 (1.83)	0
Consumption of pickled food		
Low level	37 736 (63.44)	34 589 (77.47)
High level	21 103 (35.48)	10 059 (22.53)
NA	642 (1.08)	0
Consumption of fried food		
Low level	51 893 (87.24)	41 219 (92.32)
High level	5066 (8.52)	3429 (7.68)
NA	2522 (4.24)	0
Consumption of hot food		
Low level	45 939 (77.23)	39 193 (87.78)
High level	11 294 (18.99)	5455 (12.22)
NA	2248 (3.78)	0
History of gastrointestinal tract diseases		
No	52 310 (87.94)	37 909 (84.91)
Yes	7145 (12.01)	6739 (15.09)
NA	26 (0.04)	0
Family history of any cancer		
No	48 267 (81.15)	38 951 (87.24)
Yes	11 180 (18.80)	5697 (12.76)
NA	34 (0.06)	0
No. of lesions		
0	48 669 (81.82)	37 456 (83.89)
1	8437 (14.18)	6592 (14.76)
2	1773 (2.98)	531 (1.19)
≥3	602 (1.01)	69 (0.15)
Size of distinct lesion, cm		
<1	57 080 (95.96)	42 556 (95.31)
≥1	2401 (4.04)	2092 (4.69)
Mild or moderate dysplasia		
No	56 021 (94.18)	43 686 (97.85)
Yes	3460 (5.82)	962 (2.15)

Abbreviations: BMI, body mass index (calculated as weight in kilograms divided by height in meters squared); NA, not available.

^a^ Unless otherwise indicated, data are expressed as No. (%) of participants. Percentages have been rounded and may not total 100.

### Incidence of ESCC

eTable 4 in Supplement 1 shows the incidence of ESCC. In the derivation cohort, there were 252 new cases of ESCC during 424 903.50 person-years of follow-up, and the incidence rate was 59.31 per 100 000 person-years. In the validation cohort, there were 61 new cases of ESCC during 177 094.10 person-years of follow-up, and the incidence rate was 34.45 per 100 000 person years. The median follow-up time was 6.76 (5.33-8.53) years in the derivation cohort and 4.00 (3.68-4.22) years in validation cohort.

### ESCC Risk Prediction Models

Table 2 shows the hazard ratios for both model A and model B. For model B, the most appropriate tuning parameter λ for LASSO regression was 0.00058555 when the partial likelihood binomial deviance reached its minimum value, and the final selected predictors of model B included age, sex, alcohol use status, smoking status, BMI, pickled food intake, annual household income level, history of gastrointestinal tract diseases, numbers of lesions, mild or moderate dysplasia, and size of distinct lesions. It is worth noting that interactions between age and other variables were not statistically significant. Compared with the reference value, participants who had 1 lesion had a 52% (95% CI, 4%-122%) higher risk of developing ESCC within 3 years; those with 2 lesions, 212% (95% CI, 94%-402%); and those with 3 or more lesions, 455% (95% CI, 224%-850%). Those with distinct lesions had a 62% (95% CI, 11%-131%) higher risk of developing ESCC within 3 years; those with mild or moderate dysplasia, 201% (95% CI, 106%-339%).

**Table 2.** Hazard Ratios of Risk Factors Associated With Esophageal Squamous Cell Carcinoma in the Cox Proportional Hazards Regression Model

Variable	HR (95%CI)		
	Unadjusted model	Adjusted model	
		A	B
Age, y			
40-44	1 [Reference]	1 [Reference]	1 [Reference]
45-49	2.74 (1.19-6.35)	2.53 (1.09-5.87)	2.52 (1.09-5.85)
50-54	6.48 (2.95-14.23)	5.18 (2.35-11.41)	5.08 (2.31-11.20)
55-59	7.22 (3.29-15.85)	5.06 (2.29-11.16)	4.64 (2.10-10.26)
60-64	7.77 (3.52-17.14)	4.72 (2.12-10.51)	4.38 (1.96-9.76)
65-69	10.68 (4.77-23.91)	6.00 (2.65-13.58)	5.52 (2.43-12.53)
Sex			
Female	1 [Reference]	1 [Reference]	1 [Reference]
Male	3.35 (2.55-4.39)	2.81 (2.14-3.69)	2.01 (1.42-2.85)
Smoking status			
No	1 [Reference]	NA	1 [Reference]
Yes	2.62 (2.04-3.37)	NA	1.12 (0.82-1.54)
Alcohol use			
No	1 [Reference]	NA	1 [Reference]
Yes	2.86 (2.23-3.67)	NA	1.44 (1.04-1.98)
BMI			
≥24	1 [Reference]	NA	1 [Reference]
<24	2.02 (1.56-2.61)	NA	1.35 (1.15-1.95)
Annual household income level			
High	1 [Reference]	NA	1 [Reference]
Low	1.70 (1.26-2.30)	NA	1.57 (0.99-1.83)
History of gastrointestinal tract diseases			
No	1 [Reference]	NA	1 [Reference]
Yes	1.51 (1.09-2.09)	NA	1.44 (1.13-2.18)
Consumption of pickled food			
Low level	1 [Reference]	NA	1 [Reference]
High level	1.50 (1.16-1.95)	NA	1.50 (1.10-1.87)
No. of lesions			
0	1 [Reference]	1 [Reference]	1 [Reference]
1	3.18 (2.33-4.36)	2.81 (2.14-3.69)	1.52 (1.04-2.22)
2	9.57 (6.63-13.81)	2.47 (1.8-3.40)	3.12 (1.94-5.02)
≥3	24.33 (16.34-36.24)	6.77 (4.66-9.86)	5.55 (3.24-9.50)
Size of distinct lesion, cm			
<1	1 [Reference]	NA	1 [Reference]
≥1	6.28 (4.56-8.65)	NA	1.61 (1.11-2.31)
Mild or moderate dysplasia			
No	1 [Reference]	NA	1 [Reference]
Yes	9.69 (7.37-12.74)	NA	3.01 (2.06-4.39)

Abbreviations: BMI, body mass index (calculated as weight in kilograms divided by height in meters squared); HR, hazard ratio.

### Validation of the Prediction Model

#### Discrimination Ability

Table 3 shows the discrimination performance of model A and model B. For model A in the derivation cohort, *R*^2^ was 45.12%, the D statistic was 1.86 (95% CI, 1.66-2.06), and the Harrell C statistic was 0.80 (95% CI, 0.77-0.83). In the validation cohort, *R*^2^ was 63.13%, the D statistic was 2.68 (95% CI, 2.25-3.11), and the Harrell C statistic was 0.90 (95% CI, 0.87-0.93). For model B in the derivation cohort, *R*^2^ was 51.43%, the D statistic was 2.11 (95% CI, 1.90-2.31), and the Harrell C statistic was 0.83 (95% CI, 0.81-0.86). In the validation cohort, *R*^2^ was 65.32%, the D statistic was 2.81 (95% CI, 2.39-3.22), and the Harrell C statistic was 0.91 (95% CI, 0.88-0.95).

**Table 3.** Statistics of the Performance of Developed Risk Prediction Models of Esophageal Squamous Cell Carcinoma

Statistic	Derivation cohort		Validation cohort	
	Model A^a^	Model B^b^	Model A^a^	Model B^b^
D statistic (95% CI)^c^	1.86 (1.66-2.06)	2.11 (1.90-2.31)	2.68 (2.25-3.11)	2.81 (2.39 3.22)
Harrell C statistic (95% CI)^c^	0.80 (0.77-0.83)	0.83 (0.81-0.86)	0.90 (0.87-0.93)	0.91 (0.88-0.95)
*R*^2^, %^d^	45.12	51.43	63.13	65.32

^a^ Includes variables age, sex, and number of lesions.

^b^ Includes variables age, sex, body mass index, smoking status, alcohol use, number of lesions, size of distinct lesions, and mild or moderate dysplasia.

^c^ Evaluates model discrimination ability. Higher values indicate better discrimination ability.

^d^ Interprets the variance of the model. Higher values indicate better variance.

#### Calibration Ability

eFigure 4 in Supplement 1 shows the calibration curves of model A and model B. In the derivation cohort, the intercept was 0.000 and the slope was 1.000 for model A, and the corresponding values for model B were 0.0002 and 1.000. In the validation cohort, the intercept was −0.419 and the slope was 1.712 for model A and −0.195 and 1.511, respectively, for model B. The predicted and observed risks were consistent because the points were close to the line whose slope was 1 and intercept was 0.

#### Clinical Usefulness

eFigure 5 in Supplement 1 shows that both model A and model B had a higher net benefit than treating all the entire population as a high-risk population or, otherwise, as a low-risk population. This demonstrated that models had clinical usefulness.

### Score Model

Table 4 shows the scores for age (8 for population aged 45-49 years; 14 for population aged 50-54 years; 13 for population aged 55-64 years; and 15 for population aged 65-69 years), BMI (4 for <24), sex (6 for men), smoking status (1 for smokers), alcohol use (3 for alcohol use), annual household income (3 for a low level), history of gastrointestinal tract diseases (4 for yes), pickled food consumption (3 for a high level), number of lesions (4 for 1 lesion, 10 for 2 lesions, and 15 for ≥3 lesions), size of distinct lesions (4 for lesions ≥1 cm), and mild or moderate dysplasia (10 for present). eTable 5 in Supplement 1 shows the specificity, sensitivity, and Youden index at different thresholds.

**Table 4.** Score Model Based on Model B^a^

Variable	Model B	
	Coefficient (95% CI)	Score
Age, y		
40-44	0	0
45-49	0.92 (0.09 to 1.77)	8
50-54	1.63 (0.84 to 2.42)	14
55-59	1.53 (0.74 to 2.33)	13
60-64	1.48 (0.67 to 2.28)	13
65-69	1.71 (0.89 to 2.53)	15
Sex		
Female	0	0
Male	0.70 (0.36 to 1.05)	6
Smoking status		
No	0	0
Yes	0.12 (−0.21 to 0.42)	1
Alcohol use		
No	0	0
Yes	0.36 (0.30 to 0.67)	3
BMI		
≥24	0	0
<24	0.40 (0.14 to 0.66)	4
Annual household income level		
High	0	0
Low	0.30 (−0.01 to 0.60)	3
History of gastrointestinal tract diseases		
No	0	0
Yes	0.45 (0.11 to 0.77)	4
Consumption level of pickled food		
Low level	0	0
High level	0.36 (0.10 to 0.64)	3
No. of lesions		
0	0	0
1	0.42 (0.03 to 0.80)	4
2	1.14 (0.66 to 1.61)	10
≥3	1.71 (1.18 to 2.25)	15
Size of distinct lesion, cm		
<1	0	0
≥1	0.47 (0.11 to 0.84)	4
Mild or moderate dysplasia		
No	0	0
Yes	1.10 (0.72 to 1.48)	10

Abbreviation: BMI, body mass index (calculated as weight in kilograms divided by height in meters squared).

^a^ Regression coefficients were used as weights, and the other predictors obtained their corresponding scores by dividing the smallest coefficient and rounding it to an integer. The total score can be calculated according to the prediction factors of each participant, and the risk within 3 years can be calculated.

The Figure shows the comparison performed among 4 strategies for identifying high-risk population. Strategy 1 is the current cancer screening recommended, and strategies 2, 3, and 4 used model B as an assessment tool. Results showed that model B was more sensitive.

**Figure.**
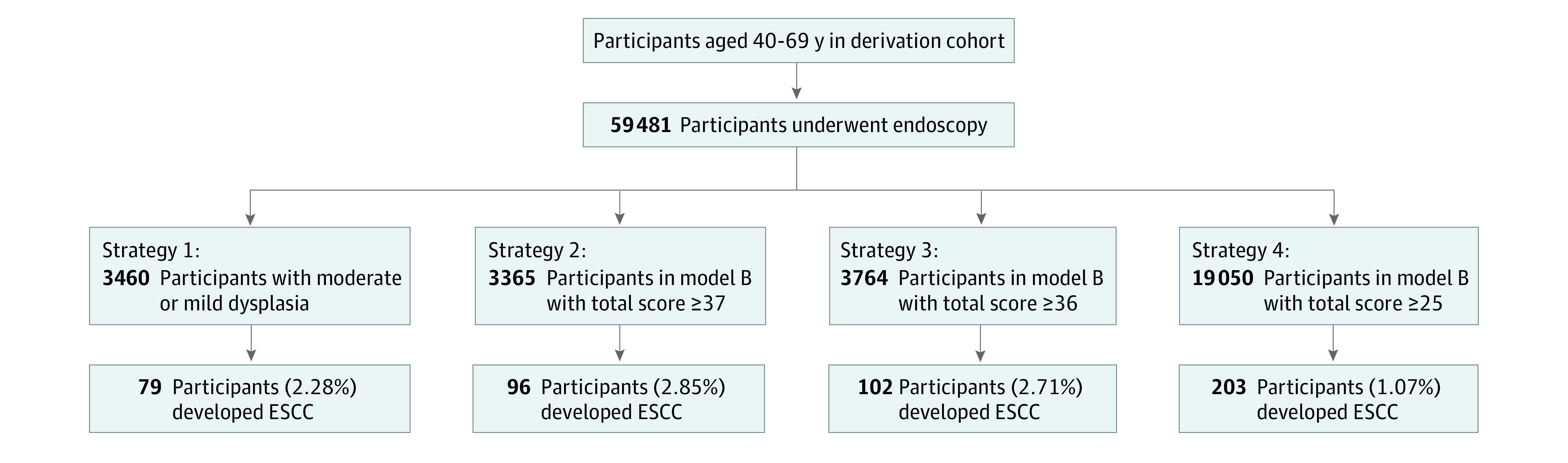
Comparison of 4 Strategies for Identifying Patients at High Risk of Developing Esophageal Squamous Cell Carcinoma (ESCC) In strategy 1, 3460 participants were followed up according to the cancer screening program. In strategy 2, 3365 participants were followed up according to their total score (≥37) based on model B. In strategy 3, 3764 participants were followed up according to their total score (≥36) based on model B. In strategy 4, 19 050 participants were followed up according to their total score (≥25) recommended based on model B.

eTable 6 in Supplement 1 shows the analysis based on complete data. eTable 7 in Supplement 1 shows the sensitivity analyses for different BMI or age classification. eTable 8 in Supplement 1 shows the performance of model A and model B for different groups. The stability of the model was verified.

## Discussion

Prediction models of ESCC based on endoscopy data provided an efficient tool for follow-up decision-making in the Chinese population in this diagnostic study. All models showed good discriminative and calibration ability. Model B had best performance, including discrimination and calibration ability, in both cohorts. These models had clinical usefulness as shown by decision curve analysis. The variables selected in this study—number of lesions, size of distinct lesions, and mild or moderate dysplasia—obtained by endoscopy could be recorded accurately by inexperienced or experienced operators better than the other features, such as the shape of lesions. The baseline characteristics and the incidence rate between derivation and verification cohorts were quite different; however, the models performed well in both data sets. Therefore, the models may be suitable for large-scale popularization and application.

According to the cancer screening process shown in eFigure 1 in Supplement 1, participants with mild dysplasia or worse lesions were followed up or received an intervention: participants with mild dysplasia were followed up after endoscopy examination in 3 or 5 years, and participants with severe dysplasia or worse had a corresponding therapeutic regimen. This follow-up plan was not precise because there was no guidance for participants with positive findings no worse than mild dysplasia. According to model B, mild or moderate dysplasia is an important risk factor for ESCC; therefore, treatment intervention for mild or moderate dysplasia could be suggested, as it may reduce the risk of ESCC. Both models suggested that participants with numbers of lesions and size of distinct lesions should be given more attention during follow-up.

This study included endoscopy-related risk factors for ESCC. Zhu et al^42^ studied the endoscopic morphological characteristics and pathological outcomes of low-grade intraepithelial neoplasia of the esophageal mucosa and found that 19 of 26 patients who progressed to high-grade intraepithelial neoplasia or invasive cancer (73.1%) had a lesion with a maximum diameter of greater than 1 cm, suggesting that a maximum diameter of greater than 1 cm was a risk factor for progression. Tanmowei et al^43^ found that low-grade intraepithelial neoplasia of the esophageal squamous epithelium is closely related to the occurrence of esophageal cancer and was related to the classification and range of esophageal endoscopic lesions. More attention should be given to patients with lesions larger than 1 cm. Several studies^12,44,45^ have shown that the risk of ESCC increases with the grade and severity of the pathological diagnosis. Wei et al^12^ found that compared with the reference group, esophagitis was not related to the occurrence of ESCC; the risk of ESCC in the group with mild to moderate dysplasia was increased; and the risk of ESCC in the group with moderate dysplasia was significantly higher. In particular, in this study, the number of lesions was used as one of the risk factors for an ESCC model that was not used before, and the discrimination of the ESCC model was perfect. In our study, the number of lesions was divided into 4 groups: 0, 1, 2, and 3 or more. Higher grades of squamous dysplasia were associated with a higher risk of ESCC.

### Strengths and Limitations

This study has strengths. First, this study established prediction models suitable for follow-up stage based on large-scale multicenter esophageal cancer screening data, which could optimize the current screening program. Second, in this study, model A and model B included the endoscopy and pathological diagnosis based on the survey data for the first time, which was of great importance for the identification of high-risk groups in the follow-up stage. They could be used in the follow-up decision-making during the screening process to optimize the screening program.

This report provides 2 ESCC risk evaluation models with good discrimination and calibration ability in both internal and external verification for different uses. These models could be used to optimize the esophageal cancer screening program and are of great significance for populations who have undergone endoscopy.

This study also has some limitations. First, the data were only from the screening data of Shandong Province and cannot represent the population aged 40 to 69 years in China. However, Shandong Province has about 100 million people, accounting for one-tenth of China's total population, which indicates that our data are somewhat representative. Data collection and analysis of other provinces will also be performed in the future. Second, although our study population was approximately 100 000, there were few new cases of ESCC. In the future, with the extension of follow-up time, the number of outcomes will increase. Third, in the model, we did not subdivide the shape and location of the lesions, which will be analyzed in the future. Fourth, for now, the model could be popularized and applied in Shandong Province as it performs well in both the derivation and validation sets. The model needs to be continually evaluated and updated for applications elsewhere or for longer periods of time.

## Conclusions

In this diagnostic study of esophageal cancer screening, we developed and validated 2 prediction models. Model A included age, sex, and number of lesions as variables, and model B included age, sex, smoking status, alcohol use status, BMI, annual household income, history of gastrointestinal tract diseases, pickled food consumption, number of lesions, size of distinct lesions, and mild or moderate dysplasia. The models had good discrimination and calibration performance and clinical usefulness. The models developed in our study were suitable for selecting high-risk populations for follow-up decision-making and were of great significance for optimizing the cancer screening process.

## Notes

Supplement 1. **eFigure 1.** Overview of the Screening Procedure of the Cancer Screening **eFigure 2.** Flow Diagram of the Study Population **eFigure 3.** Cumulative Incidence Curves for Endoscopic-Related Risk Factors **eFigure 4.** Observed Proportion of ESCC and Predicted Risk of ESCC Within 3 Years in the Derivation Cohort and Validation Cohort of Model A and Model B **eFigure 5.** Decision Curves of Model A and Model B in the Derivation Cohort and Validation Cohort **eTable 1.** Prediction Models for Esophageal Squamous Cell Carcinoma Developed in Previous Studies **eTable 2.** Description of Candidate Variables **eTable 3.** Baseline Characteristics of Participants Split by the End Point in Derivation Cohort and Validation Cohort **eTable 4.** Incidence Rates of ESCC per 100 000 Person-Years in Derivation Cohort and Validation Cohort **eTable 5.** Sensitivity, Specificity, and Other Measures at Different Thresholds of Risk for Score Model Based on Model B **eTable 6.** Hazard Ratio of Risk Factors Associated With ESCC in the Multivariable Cox Model (Use Complete Data) **eTable 7.** Statistics of the Performance of Developed Risk-Prediction Models for Different Classification (Age and BMI) (Use Complete Data) **eTable 8.** Statistics of the Performance of Developed Risk-Prediction Models for Different Groups (Use Complete Data)
eReferences

Supplement 2.
Data Sharing Statement
