# Supplementary material for: Development and Validation of Esophageal Squamous Cell Carcinoma Risk Prediction Models Based on an Endoscopic Screening Program
Source: JAMA Netw Open. 2023 Jan 26;6(1):e2253148. doi: 10.1001/jamanetworkopen.2022.53148 (PMC9880791; doi:10.1001/jamanetworkopen.2022.53148)
Supplement: Supplement 1. — eFigure 1. Overview of the Screening Procedure of the Cancer Screening eFigure 2. Flow Diagram of the Study Population eFigure 3. Cumulative Incidence Curves for Endoscopic-Related Risk Factors eFigure 4. Observed Proportion of ESCC and Predicted Risk of ESCC Within 3 Years in the Derivation Cohort and Validation Cohort of Model A and Model B eFigure 5. Decision Curves of Model A and Model B in the Derivation Cohort and Validation Cohort eTable 1. Prediction Models for Esophageal Squamous Cell Carcinoma Developed in Previous Studies eTable 2. Description of Candidate Variables eTable 3. Baseline Characteristics of Participants Split by the End Point in Derivation Cohort and Validation Cohort eTable 4. Incidence Rates of ESCC per 100 000 Person-Years in Derivation Cohort and Validation Cohort eTable 5. Sensitivity, Specificity, and Other Measures at Different Thresholds of Risk for Score Model Based on Model B eTable 6. Hazard Ratio of Risk Factors Associated With ESCC in the Multivariable Cox Model (Use Complete Data) eTable 7. Statistics of the Performance of Developed Risk-Prediction Models for Different Classification (Age and BMI) (Use Complete Data) eTable 8. Statistics of the Performance of Developed Risk-Prediction Models for Different Groups (Use Complete Data) eReferences [file jamanetwopen-e2253148-s001.pdf]

Supplementary Online Content

Han J, Guo X, Zhao L, et al. Development and validation of esophageal squamous cell carcinoma risk prediction models based on an endoscopic screening program. *JAMA Netw Open*. 2023;6(1):e2253148. doi:10.1001/jamanetworkopen.2022.53148

eFigure 1. Overview of the Screening Procedure of the Cancer Screening

eFigure 2. Flow Diagram of the Study Population

eFigure 3. Cumulative Incidence Curves for Endoscopic-Related Risk Factors

eFigure 4. Observed Proportion of ESCC and Predicted Risk of ESCC Within 3 Years in the Derivation Cohort and Validation Cohort of Model A and Model B

eFigure 5. Decision Curves of Model A and Model B in the Derivation Cohort and Validation Cohort

eTable 1. Prediction Models for Esophageal Squamous Cell Carcinoma Developed in Previous Studies

eTable 2. Description of Candidate Variables

eTable 3. Baseline Characteristics of Participants Split by the End Point in Derivation Cohort and Validation Cohort

eTable 4. Incidence Rates of ESCC per 100 000 Person-Years in Derivation Cohort and Validation Cohort

eTable 5. Sensitivity, Specificity, and Other Measures at Different Thresholds of Risk for Score Model Based on Model B

eTable 6. Hazard Ratio of Risk Factors Associated With ESCC in the Multivariable Cox Model (Use Complete Data)

eTable 7. Statistics of the Performance of Developed Risk-Prediction Models for Different Classification (Age and BMI) (Use Complete Data)

eTable 8. Statistics of the Performance of Developed Risk-Prediction Models for Different Groups (Use Complete Data)

eReferences

This supplementary material has been provided by the authors to give readers additional information about their work.

© 2023 Han J et al. *JAMA Network Open*.

eFigure 1. Overview of the Screening Procedure of the Cancer Screening

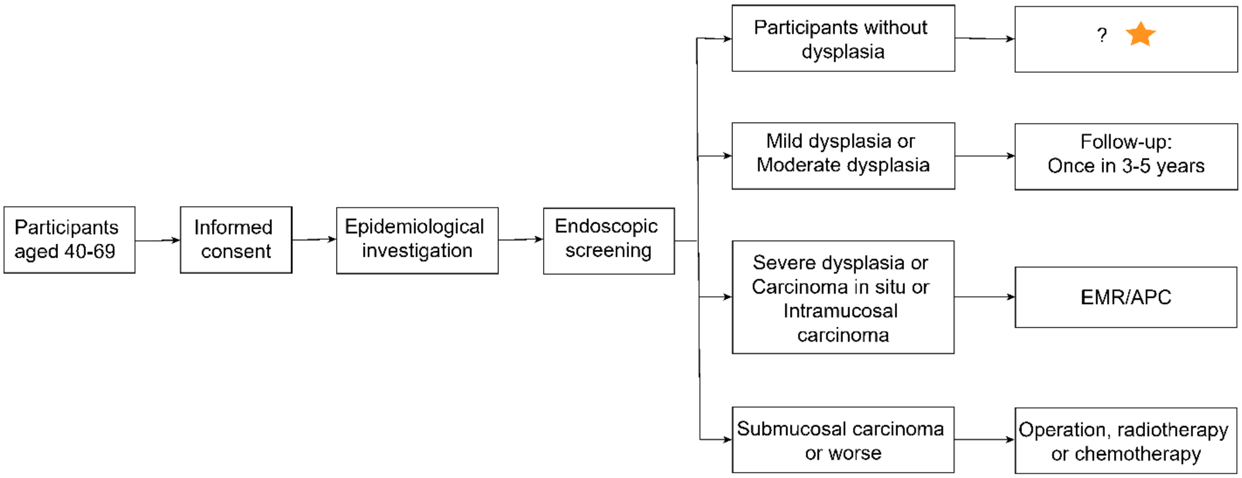

APC: Argon Plasma Coagulation; EMR: endoscopic mucosal resection

©2023 Han J et al. *JAMA Network Open*.

eFigure 2. Flow Diagram of the Study Population

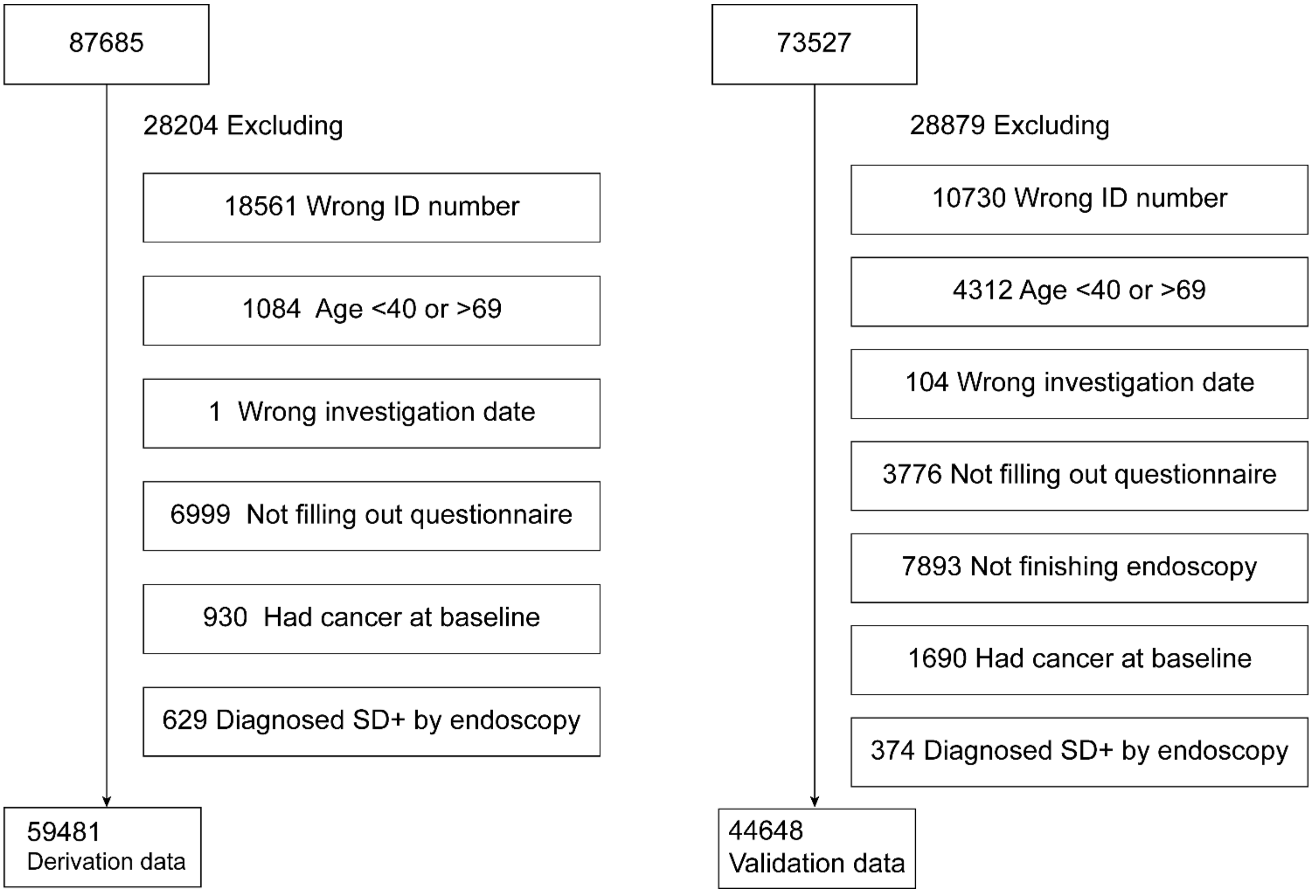

©2023 Han J et al. *JAMA Network Open*.

eFigure 3. Cumulative Incidence Curves for Endoscopic-Related Risk Factors

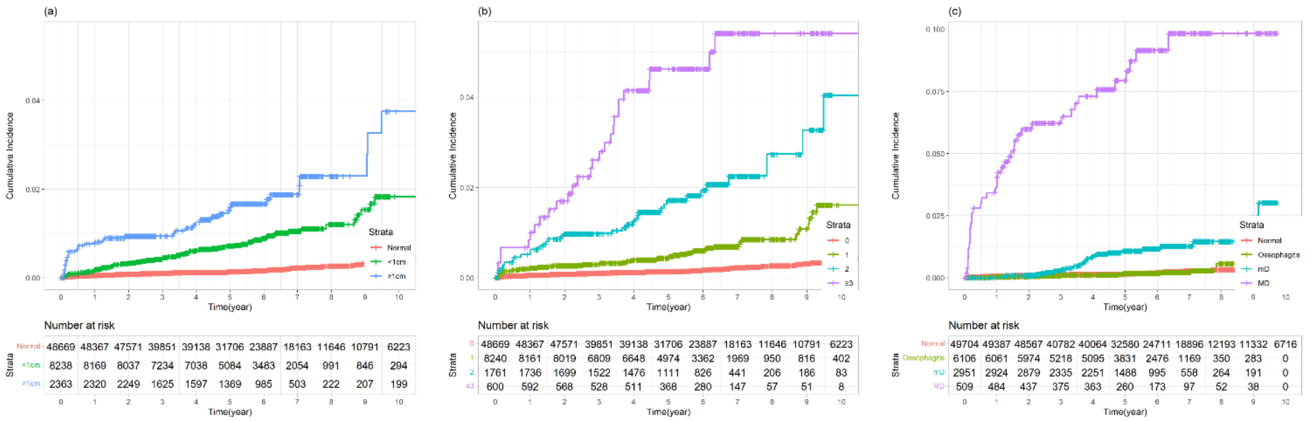

(a): Cumulative incidence curves of groups with different lesion size. Normal: with no lesions; (b): Cumulative incidence curves of groups with different number of lesions. (c): Cumulative incidence curves of groups with different pathologic diagnosis. In above image, mD represented mild dysplasia and MD represented moderate dysplasia; The unit of time is year and the unit of cumulative incidence is %.

© 2023 Han J et al. *JAMA Network Open*.

eFigure 4. Observed Proportion of ESCC and Predicted Risk of ESCC Within 3 Years in the Derivation Cohort and Validation Cohort of Model A and Model B

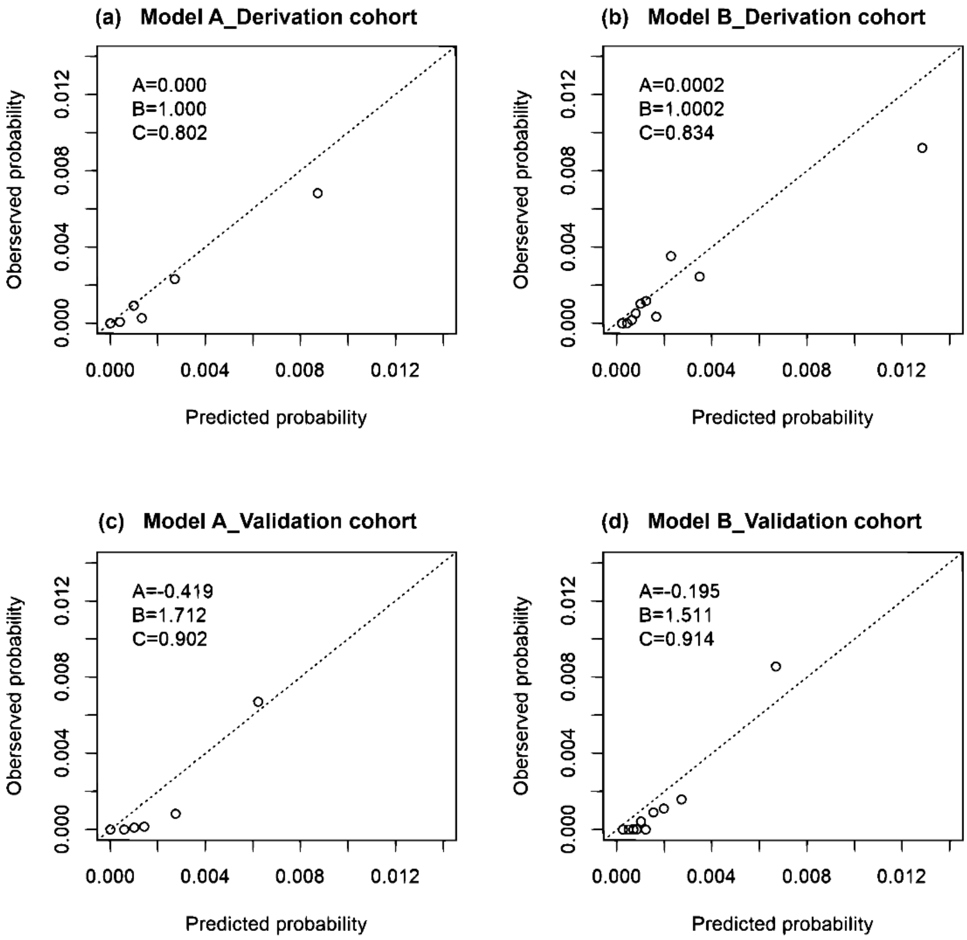

A represents the intercept; B represents the slope; C represents c-statistic.

© 2023 Han J et al. *JAMA Network Open*.

eFigure 5. Decision Curves of Model A and Model B in the Derivation Cohort and Validation Cohort

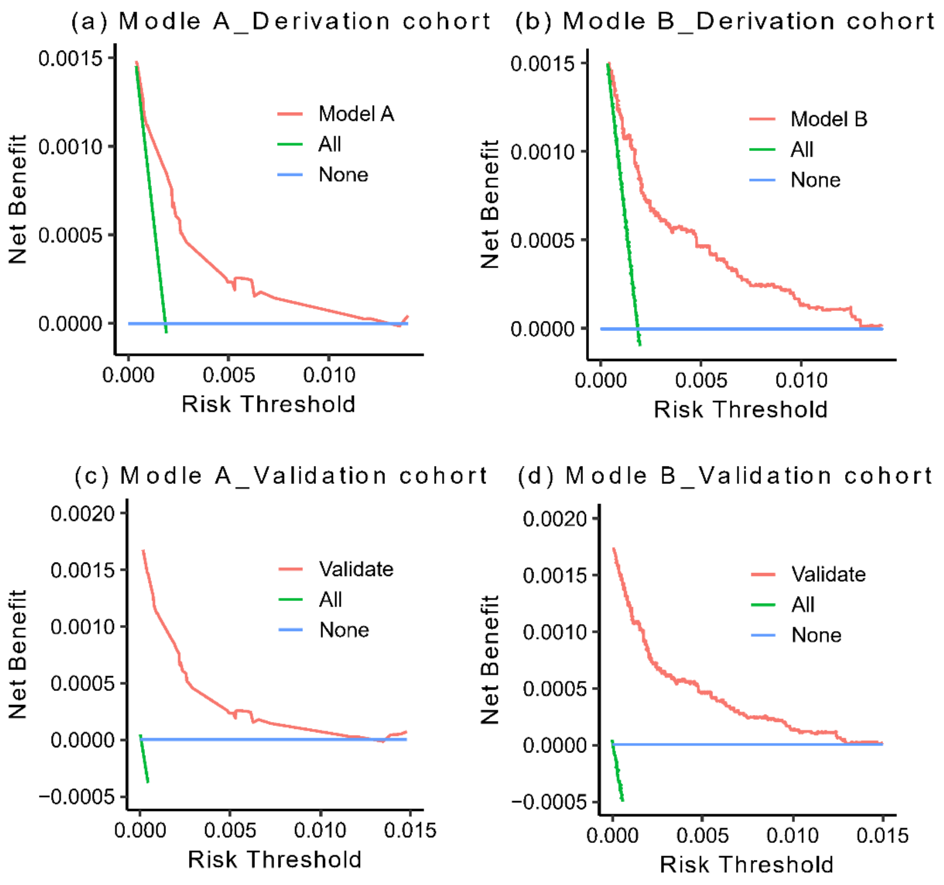

The horizontal axis of this picture was threshold probability. The ordinate axis was the net benefit after the advantages subtracted by the disadvantages.

© 2023 Han J et al. *JAMA Network Open*.

eTable 1. Prediction Models for Esophageal Squamous Cell Carcinoma Developed in Previous Studies

Authors	Setting	Predictors	Derivation performance AUC (95%CI)	Validation performance AUC (95%CI)
Chen et al. [1]	Cohort	age, sex, family history of upper gastrointestinal cancer, smoking status, alarming symptoms of retrosternal pain, back pain or neck pain, consumption of salted food and fresh fruits and disease history of peptic ulcer or esophagitis	0.81(0.78-0.83)	NA
Wang et al. [2]	Cohort	age, sex, smoking, alcohol, and BMI	0.76(0.58–0.93)	0.70(0.64–0.75)
Wang et al. [3]	Case-control	age, sex, tobacco smoking, alcohol overconsumption, education, duration of living with a partner, and place of residence during childhood g	0.81(0.77-0.84）	NA
		Age, sex, smoking, drinking	0.79(0.75-0.82)	NA
Etemadi et al. [4]	Case-control	age, ethnicity, tobacco smoking, opium use, education, marital status, oral health, family history, tea temperature, and water source, unintentional weight loss	0.77(0.74-0.80)	NA
Shen et al. [5]	Case-control	age, sex, tobacco smoking, alcohol drinking, education, and dietary habits (intake of hot food, intake of pickled/salted food, and intake of fresh fruit)	0.79(0.75-0.82)	NA
Yang et al. [6]	Case-control	Women: education, family wealth score, BMI, adult height, tooth brushing times, missing and filled teeth number, and family history of esophageal cancer	0.76 (0.73–0.79)	NA
		Men: age group, education, family wealth score, adult height, frequency of tooth brushing, missing and filled teeth, smoking pack-years, alcohol drinking intensity, tea drinking temperature, and family	0.81(0.79, 0.84)	NA

© 2023 Han J et al. *JAMA Network Open*.

		history of esophageal cancer		
Liu et al. [7]	Case-control	60 years of age or younger: age closer to 60 years, use of coal or wood as a main source of cooking fuel, body mass index of 22 kg/m2 or less, unexplained epigastric pain, and rapid ingestion of meals	0.795(0.736-0.854)	NA
		older than 60 years: age, family history of ESCC, cigarette smoking, body mass index of 22 kg/m2 or less, pesticide exposure, irregular eating habits, intake of high temperature foods, rapid ingestion of meals, and ingestion of leftover food in summer months	0.681(0.618-0.743)	NA
Yokoyama et al. [8	Case-control	past and current alcohol flushing tendency, drinking, smoking, and intake of vegetables and fruits	NA	NA
Han et al. [9]	cohort	age, sex, alcohol drinking status, BMI, tea drinking status, and fresh fruit	0.792 (0.761,0.822)	0.773 (0.736,0.811)
Liu er al. [10]	Case-control	age and its quadratic term, family history of ESCC, low body mass index (≤22 kg/m2), use of coal or wood as main fuel for cooking, eating rapidly, and ingestion of leftover food.	0·75 (0·72-0·79)	0·71 (0·65-0·78)

Abbreviations: BMI, body mass index; ESCC, esophageal squamous cell carcinoma; AUC, aera under the curve.

© 2023 Han J et al. *JAMA Network Open*.

eTable 2. Description of Candidate Variables

variable	Variable types	Definitions
Age	categorical variable	40-44; 45-49; 50-54; 55-59; 60-64; 65-69
Sex	categorical variable	Male； Female
BMI	categorical variable	≥24; <24
Source of drinking water	categorical variable	Treated water; Untreated water
Annual household income	categorical variable	≥20000 yuan; <20000 yuan
Smoking status	categorical variable	Yes; No
Drinking status	categorical variable	Yes; No
Tea drinking	categorical variable	Yes; No
Fresh fruit	categorical variable	High level; Low level
Pickled food	categorical variable	High level; Low level
Fried food	categorical variable	High level; Low level
Hot food	categorical variable	High level; Low level
History of gastrointestinal diseases	categorical variable	Yes; No
Family history of any cancer	categorical variable	Yes; No
NLs	categorical variable	Yes; No
DL	categorical variable	≥1cm； others
MD	categorical variable	MD; others

Abbreviations: BMI, body mass index; NLs, Number of lesions (suspicious lesions or positive findings); DL, distinct lesions(>=1cm); MD, moderate or mild dysplasia. High level, ≥twice a week. Low level, <twice a week.

© 2023 Han J et al. *JAMA Network Open*.

eTable 3. Baseline Characteristics of Participants Split by the End Point in Derivation Cohort and Validation Cohort

Variable	Derivation cohort No. (%)		Validation cohort No. (%)	
	ESCC free (n=59229)	ESCC cases (n=252)	ESCC free (n=44587)	ESCC cases (n=61)
Age[ab], mean (SD), y	53.82(7.64)	57.12(6.78)	54.94(7.60)	60.95(5.61)
Age[ab]				
40-44	7750(13.08)	7(2.78)	4093(9.17)	0(0.00)
45-49	11747(19.83)	26(10.32)	8049(18.03)	1(1.64)
50-54	12642(21.34)	61(24.21)	10127(22.68)	7(11.48)
55-59	11218(18.94)	61(24.21)	7862(17.61)	17(27.87)
60-64	10014(16.91)	55(21.83)	8500(19.04)	18(29.51)
65-69	5858(9.89)	42(16.67)	5956(13.34)	18(29.51)
Sex[ab]				
Female	34752(58.67)	75(29.76)	23884(53.49)	7(11.48)
Male	24477(41.33)	177(70.24)	20703(46.37)	54(88.52)
Annual household income[ab]				
High level	20283(34.25)	56(22.22)	23311(52.21)	17(27.87)
Low level	38934(65.73)	195(77.38)	21276(47.65)	44(72.13)
NA	12(0.02)	1(0.40)	0	0
Source of drinking water[a]				
Treated water	11762(19.86)	30(11.91)	37873(84.83)	50(81.97)
Untreated water	44836(75.7)	218(86.51)	4078(9.13)	10(16.39)
NA	2631(4.44)	4(1.58)	2636(5.9)	1(1.64)
Smoking status[ab]				
No	46657(78.77)	143(56.75)	36949(82.76)	37(60.66)
Yes	12547(21.18)	108(42.86)	7638(17.11)	24(39.34)
NA	25(0.04)	1(0.39)	0	0
Drinking status[ab]				
No	45724(77.2)	131(51.98)	36846(82.53)	33(54.10)
Yes	13485(22.77)	120(47.62)	7741(17.34)	28(45.90)
NA	20(0.03)	1(0.40)	0	0
Tea drinking[b]				
No	16372(27.64)	61(24.21)	29962(67.11)	31(50.82)
Yes	42837(72.32)	190(75.40)	14625(32.76)	30(49.18)
NA	20(0.03)	1(0.39)	0	0
BMI[a]				
≥24	33618(56.76)	95(37.70)	23601(52.86)	21(34.43)
<24	25564(43.16)	156(61.90)	20986(47)	40(65.57)
NA	47(0.08)	1(0.40)	0	0
Fresh fruit[ab]				
High level	40534(68.44)	142(56.35)	33124(74.19)	31(50.82)
Low level	17625(29.76)	89(35.32)	11463(25.67)	30(49.18)
NA	1070(1.81)	21(8.33)	0	0
Pickled food[a]				
Low level	37621(63.52)	115(45.64)	34545(77.37)	44(72.13)
High level	20974(35.41)	129(51.19)	10042(22.49)	17(27.87)
NA	634(1.07)	8(3.17)	0	0
Fried food				
Low level	51700(87.29)	193(76.59)	41162(92.19)	57(93.44)

© 2023 Han J et al. *JAMA Network Open*.

High level	5047(8.52)	19(7.54)	3425(7.67)	4(6.56)
NA	2482(4.19)	40(15.87)	0	0
Hot food				
Low level	45775(77.28)	164(65.08)	39140(87.66)	53(86.89)
High level	11243(18.98)	51(20.24)	5447(12.2)	8(13.11)
NA	2211(3.73)	37(14.68)	0	0
History of gastrointestinal diseases[a]				
No	52104(87.97)	206(81.75)	37854(84.78)	55(90.16)
Yes	7100(11.99)	45(17.86)	6733(15.08)	6(9.84)
NA	25(0.04)	1(0.39)	0	0
Family history of any cancer	(0)			
No	48073(81.16)	194(76.98)	38895(87.11)	56(91.80)
Yes	11123(18.78)	57(22.62)	5692(12.75)	5(8.20)
NA	33(0.06)	1(0.40)	0	0
NLs[ab]				
0	48545(81.96)	124(49.21)	37443(83.86)	13(21.30)
1	8378(14.15)	59(23.41)	6554(14.68)	38(62.30)
2	1735(2.93)	38(15.08)	525(1.18)	6(9.84)
≥3	571(0.96)	31(12.30)	65(0.15)	4(6.56)
DL[ab]				
<1cm	56875(96.03)	205(81.35)	42533(95.26)	23(37.70)
≥1cm	2354(3.97)	47(18.65)	2054(4.6)	38(62.30)
MD[ab]				
No	55848(94.29)	173(68.65)	43636(97.73)	50(81.97)
Yes	3381(5.71)	79(31.35)	951(2.13)	11(18.03)

Abbreviations: ESCC, esophageal squamous cell carcinoma; BMI, body mass index; NLs, Number of lesions (suspicious lesions or positive findings); MD, mild or moderate dysplasia; DL, distinct lesions(>=1cm). [a] indicates that risk factors were statistically different between the two groups(P<0.05) in derivation cohort; [b] indicates that risk factors were statistically different between the two groups(P<0.05) in validation cohort;

© 2023 Han J et al. *JAMA Network Open*.

eTable 4. Incidence Rates of ESCC per 100 000 Person-Years in Derivation Cohort and Validation Cohort

Group	Derivation cohort		Validation cohort	
	Incident cases	Person years (Rate per 100000 person years)	Incident cases	Person years (Rate per 100000 person years)
Total	252	424903.50(59.31)	61	177094.10(34.45)
Sex				
Women	75	249519.10(30.06)	7	94566.81(7.40)
Men	177	175384.40(100.92)	54	82527.28(65.43)

Abbreviations: ESCC, esophageal squamous cell carcinoma.

© 2023 Han J et al. *JAMA Network Open*.

eTable 5. Sensitivity, Specificity, and Other Measures at Different Thresholds of Risk for Score Model Based on Model B

Risk score	Hr	Sensitivity	Specificity	Youden's Index	AR	PPV	NPV	+LR	-LR	NNBS
17	0.699	0.991	0.301	0.292	0.302	0.003	1.000	1.418	0.031	388.766
18	0.638	0.981	0.363	0.344	0.364	0.003	1.000	1.540	0.051	358.000
19	0.594	0.963	0.407	0.370	0.408	0.003	1.000	1.624	0.091	339.442
20	0.556	0.963	0.445	0.408	0.446	0.003	1.000	1.734	0.083	317.990
21	0.490	0.907	0.511	0.418	0.511	0.003	1.000	1.854	0.181	297.531
22	0.443	0.889	0.558	0.447	0.558	0.004	1.000	2.010	0.199	274.448
23	0.408	0.870	0.593	0.463	0.594	0.004	1.000	2.139	0.219	258.011
24	0.364	0.824	0.637	0.461	0.637	0.004	0.999	2.271	0.276	243.124
25	0.320	0.824	0.681	0.505	0.681	0.005	1.000	2.579	0.259	214.124
26	0.287	0.806	0.714	0.520	0.715	0.005	1.000	2.821	0.272	195.885
27	0.254	0.741	0.747	0.487	0.747	0.005	0.999	2.924	0.347	189.000
28	0.221	0.685	0.780	0.465	0.779	0.006	0.999	3.109	0.404	177.851
29	0.195	0.639	0.805	0.444	0.805	0.006	0.999	3.283	0.448	168.449
30	0.171	0.583	0.830	0.413	0.830	0.006	0.999	3.432	0.502	161.206
31	0.141	0.556	0.860	0.415	0.859	0.007	0.999	3.966	0.517	139.600
32	0.120	0.528	0.881	0.409	0.881	0.008	0.999	4.444	0.536	124.719
33	0.105	0.519	0.896	0.414	0.895	0.009	0.999	4.962	0.538	111.786
34	0.088	0.481	0.913	0.394	0.912	0.010	0.999	5.506	0.568	100.846
35	0.072	0.454	0.929	0.382	0.928	0.011	0.999	6.359	0.588	87.449
36	0.063	0.454	0.937	0.391	0.937	0.013	0.999	7.245	0.583	76.878

Abbreviations: Hr, ratio of high risk participants; AR, accuracy rate; PPV, positive predictive value; NPV, negative predictive value; +LR, positive likelihood ratio; -LR, negative likelihood ratio; NNBS, number needed to be screened

© 2023 Han J et al. *JAMA Network Open*.

eTable 6. Hazard Ratio of Risk Factors Associated With ESCC in the Multivariable Cox Model (Use Complete Data)

Variable	Model A	Model B
	Adjusted HR (95%CI)	Adjusted HR (95%CI)
Age		
40-44	1	1
45-49	2.53(1.10,5.84)	2.49(1.08,5.75)
50-54	5.18(2.36,11.36)	4.60(2.09,10.14)
55-59	5.06(2.30,11.12)	4.58(2.08,10.09)
60-64	4.72(2.13,10.46)	4.18(1.87,9.29)
65-69	6.00(2.66,13.52)	5.09(2.25,11.54)
Sex		
Female	1	1
Male	2.81(2.14,3.69)	1.92(1.35,2.73)
NLs		
0	1	1
1	2.47(1.80,3.39)	1.49(1.01,2.20)
2	6.77(4.66,9.84)	2.87(1.76,4.68)
≥3	15.47(10.29,23.27)	5.45(3.18,9.40)
Smoking status		
No	-	1
Yes	-	1.15(0.83,1.58)
Drinking status		
No	-	1
Yes	-	1.42(1.03,1.97)
BMI		
≥24	-	1
<24	-	1.47(1.13,1.92)
Annual household income		
High level	-	1
Low level	-	1.38(1.01,1.88)
History of gastrointestinal diseases		
No	-	1
Yes	-	1.60(1.15,2.23)
Pickled food		
Low level	-	1
High level	-	1.44(1.11,1.88)
DL		
<1cm	-	1
≥1cm	-	1.64(1.13,2.37)
MD		
No	-	1
Yes	-	3.08(2.09,4.53)

Abbreviations: BMI, body mass index; NLs, Number of lesions (suspicious lesions or positive findings); DL, distinct lesions(>=1cm); MD, moderate or mild dysplasia.

eTable 7. Statistics of the Performance of Developed Risk-Prediction Models for Different Classification (Age and

© 2023 Han J et al. *JAMA Network Open*.

BMI) (Use Complete Data)

	Statistic	Derivation		Validation	
		Model A	Model B	Model A	Model B
Models in the manuscript	D statistic	1.86(1.66, 2.06)	2.09(1.88, 2.30)	2.68(2.25, 3.11)	2.83(2.41, 3.24)
	Harrell's C	0.80(0.77, 0.83)	0.83(0.81, 0.86)	0.90(0.87, 0.93)	0.92(0.88, 0.95)
	$\boldsymbol{R^2}$(%)	45.12	51.05	63.13	65.59
BMI					
>=25; <25	D statistic	1.86(1.66, 2.06)	2.09(1.88, 2.29)	2.68(2.25, 3.11)	2.82(2.40, 3.23)
	Harrell's C	0.80(0.77, 0.83)	0.83(0.81, 0.86)	0.90(0.87, 0.93)	0.92(0.88, 0.95)
	$\boldsymbol{R^2}$(%)	45.12	50.93	63.13	65.42
>22.9; <=22.9	D statistic	1.86(1.66, 2.06)	2.09(1.89,2.30)	1.86(1.66, 2.06)	2.80(2.38,3.22)
	Harrell's C	0.80(0.77, 0.83)	0.84(0.81,0.86)	0.80(0.77, 0.83)	0.92(0.88,0.95)
	$\boldsymbol{R^2}$(%)	45.12	51.14	45.12	65.18
Age					
continuous variable	D statistic	1.83(1.64,2.03)	2.09(1.88, 2.30)	2.79(2.37,3.22)	2.85(2.43,3.26)
	Harrell's C	0.80(0.77, 0.83)	0.83(0.80, 0.85)	0.91(0.87,0.94)	0.91(0.87, 0.95)
	$\boldsymbol{R^2}$(%)	44.54	51.05	65.10	65.96
40-49;50-59;60-69	D statistic	1.80(1.60,2.00)	2.09(1.88,2.29)	2.75(2.31,3.18)	2.83(2.41,3.25)
	Harrell's C	0.79(0.76,0.82)	0.83(0.80,0.86)	0.89(0.86,0.93)	0.91(0.88,0.95)
	$\boldsymbol{R^2}$(%)	44.60	50.97	64.31	65.70

Abbreviations: Model A included variables age, sex and NLs; Model B included variables age, sex, BMI, smoking status, drinking status, NLs, DL and MD. NLs, Number of lesions (suspicious lesions or positive findings); DL, distinct lesions(>=1cm); MD, moderate or mild dysplasia. D statistic and Harrell's C, evaluate model discrimination ability, higher values mean better discrimination ability; R^2, the variance the model interpretated, higher value means better.

eTable 8. Statistics of the Performance of Developed Risk-Prediction Models for Different Groups (Use Complete Data)

Group	Statistic	Derivation		Validation	
		Model A	Model B	Model A	Model B
DL<1cm	D statistic	1.81(1.59,2.03)	2.05(1.83,2.28)	2.14(1.47,2.81)	1.87(1.22,2.53)
	Harrell's C	0.80(0.76,0.83)	0.82(0.80,0.85)	0.87(0.82,0.92)	0.85(0.79,0.91)
	$\boldsymbol{R^2}$(%)	43.91	50.19	52.15	45.60
MD: No	D statistic	1.41(1.18,1.65)	1.70(1.46,1.95)	2.75(2.28,3.22)	2.71(2.27,3.16)
	Harrell's C	0.76(0.72,0.79)	0.80(0.76,0.83)	0.92(0.88,0.95)	0.90(0.86,0.94)
	$\boldsymbol{R^2}$(%)	32.21	40.95	64.32	63.71
NLs=0	D statistic	1.38(1.07,1.68)	1.53(1.25,1.81)	2.45(1.27,3.63)	1.57(0.73,2.41)
	Harrell's C	0.75(0.71,0.79)	0.78(0.75,0.82)	0.86(0.81,0.91)	0.83(0.76,0.89)
	$\boldsymbol{R^2}$(%)	31.15	35.77	58.83	37.03
Age≥50	D statistic	1.49(1.28,1.71)	1.80(1.57,2.02)	2.30(1.87,2.73)	2.54(2.12,2.96)
	Harrell's C	0.74(0.70,0.78)	0.79(0.75,0.82)	0.86(0.81,0.90)	0.89(0.85,0.93)
	$\boldsymbol{R^2}$(%)	34.73	43.50	55.82	60.61

Abbreviations: Model A included variables age, sex and NLs; Model B included variables age, sex, BMI, smoking status, drinking status, NLs, DL and MD. NLs, Number of lesions (suspicious lesions or positive findings); DL, distinct lesions(>=1cm); MD, moderate or mild dysplasia. D statistic and Harrell's C, evaluate model discrimination ability, higher values mean better discrimination ability; R^2, the variance the model interpretated, higher value means better.

© 2023 Han J et al. *JAMA Network Open*.

eReferences

[1] Chen W, Li H, Ren J, et al. Selection of high-risk individuals for esophageal cancer screening: A prediction model of esophageal squamous cell carcinoma based on a multicenter screening cohort in rural China[J]. Int J Cancer, 2021,148(2):329-339.

[2] Wang Q L, Ness-Jensen E, Santoni G, et al. Development and Validation of a Risk Prediction Model for Esophageal Squamous Cell Carcinoma Using Cohort Studies[J]. Am J Gastroenterol, 2020,116(4):683-691.

[3] Wang Q L, Lagergren J, Xie S H. Prediction of individuals at high absolute risk of esophageal squamous cell carcinoma[J]. Gastrointest Endosc, 2019,89(4):726-732.

[4] Etemadi A, Abnet C C, Golozar A, et al. Modeling the risk of esophageal squamous cell carcinoma and squamous dysplasia in a high risk area in Iran[J]. Arch Iran Med, 2012,15(1):18-21.

[5] Shen Y, Xie S, Zhao L, et al. Estimating Individualized Absolute Risk for Esophageal Squamous Cell Carcinoma: A Population-Based Study in High-Risk Areas of China[J]. Front Oncol, 2020,10:598603.

[6] Yang X, Suo C, Zhang T, et al. A nomogram for screening esophageal squamous cell carcinoma based on environmental risk factors in a high-incidence area of China: a population-based case-control study[J]. BMC Cancer, 2021,21(1):343.

[7] Liu M, Liu Z, Cai H, et al. A Model To Identify Individuals at High Risk for Esophageal Squamous Cell Carcinoma and Precancerous Lesions in Regions of High Prevalence in China[J]. Clin Gastroenterol Hepatol, 2017,15(10):1538-1546.

[8] Yokoyama A, Oda J, Iriguchi Y, et al. A health-risk appraisal model and endoscopic mass screening for esophageal cancer in Japanese men[J]. Dis Esophagus, 2013,26(2):148-153.

[9] Han J, Wang L, Zhang H, et al. Development and Validation of an Esophageal Squamous Cell Carcinoma Risk Prediction Model for Rural Chinese: Multicenter Cohort Study. Front Oncol. 2021 Aug 30;11:729471.

[10] Liu M, Zhou R, Liu Z, et al. Update and validation of a diagnostic model to identify prevalent malignant lesions in esophagus in general population. EClinicalMedicine. 2022 Apr 16;47:101394.

© 2023 Han J et al. *JAMA Network Open*.
