# Supplementary material for: Development and Validation of Esophageal Squamous Cell Carcinoma Risk Prediction Models Based on an Endoscopic Screening Program
Source: JAMA Netw Open. 2023 Jan 26;6(1):e2253148. doi: 10.1001/jamanetworkopen.2022.53148 (PMC9880791; doi:10.1001/jamanetworkopen.2022.53148)
Supplement: Supplement 2. — Data Sharing Statement [file jamanetwopen-e2253148-s002.pdf]

Data Sharing Statement

Han. Development and Validation of Esophageal Squamous Cell Carcinoma Risk Prediction Models Based on an Endoscopic Screening Program. *JAMA Netw Open*. Published January 26, 2023. doi:10.1001/jamanetworkopen.2022.53148

Data
Data available: No

Additional Information
Explanation for why data not available: The data used in this research and related data can be obtained upon reasonable request from the corresponding author.
